# *Cryptosporidium* Rabbit Genotype, a Newly Identified Human Pathogen

**DOI:** 10.3201/eid1505.081419

**Published:** 2009-05

**Authors:** Rachel M. Chalmers, Guy Robinson, Kristin Elwin, Stephen J. Hadfield, Lihua Xiao, Una Ryan, Deborah Modha, Catherine Mallaghan

**Affiliations:** National Public Health Service for Wales, Swansea, Wales, UK (R.M. Chalmers, G. Robinson, K. Elwin, S.J. Hadfield); Centers for Disease Control and Prevention, Atlanta, Georgia, USA (L. Xao); Murdoch University, Murdoch, Western Australia, Australia (U. Ryan); Health Protection Agency East Midlands South, Leicester, UK (D. Modha, C. Mallaghan)

**Keywords:** Zoonoses, parasites, Cryptosporidium, rabbit, genotype, human, pathogen, waterborne, outbreak, letter

**To the Editor:** Most human cases of cryptosporidiosis are caused by *Cryptosporidium parvum* or *C*. *hominis*, but pathogenicity of some unusual *Cryptosporidium* species/genotypes is uncertain (*1*). In July 2008, an outbreak caused by *Cryptosporidium* rabbit genotype was linked to consumption of tap water in Northamptonshire, England (*2*). On June 23 and 24, *Cryptosporidium* oocysts were detected by operational monitoring of treated water at a surface water treatment works. A precautionary boil-water notice was implemented on June 25.

Enhanced surveillance for cases was established by a health protection team on June 25 in the affected area. Eight single-well immunofluorescent microscopy slides, on which oocysts were detected by water company sampling of the distribution system, were sent to the UK Cryptosporidium Reference Unit, Swansea, for typing. Slides contained 49–259 oocysts. Coverslips were removed after softening the seal with nail polish remover. Fixed material was resuspended from the slides by thorough scraping of the entire well with a pipette tip twice with 50 μL lysis buffer AL (QIAGEN, Crawley, UK) and twice with 50 μL reverse osmosis water to a final volume of 200 μL. Oocysts were disrupted in 3 dry ice/methanol freeze-thaw cycles, and DNA was extracted by using the QIAamp DNA Mini Kit (QIAGEN), which involved digestion with proteinase K in lysis buffer AL at 56°C for 30 min, purification in a spin column, elution in 50 μL buffer AE, and storage at –20°C (*3*).

*Cryptosporidium* oocysts were also detected by direct immunofluorescent antibody test (IFAT) (Crypto-Cel; TCS Biosciences, Buckingham, UK) in large bowel contents from a rabbit carcass removed by the water company from a tank at the water treatment works. Oocysts were separated from fecal debris by flotation, resuspended in reverse osmosis water (*4*), and processed as above.

*Cryptosporidium* species were identified by bidirectional sequencing of PCR products generated by nested PCR for the small subunit (SSU) rRNA gene (*5*) from 4 DNA aliquots of each sample. SSU rDNA sequences from 7 water samples, containing 49–197 oocysts, and the rabbit isolate were homologous with isolates from rabbits in the People’s Republic of China (*6*) and the Czech Republic (*7*) (GenBank accession nos. AY120901 and AY273771, respectively) (Appendix Table). One sample from 1,391 L of water contained 259 oocysts but was not amplified. Other cryptosporidia were not identified.

Human stool samples from 34 local laboratory-identified cases of cryptosporidiosis in the affected area were sent to the UK Cryptosporidium Reference Unit for typing. To differentiate rabbit genotype from *C*. *hominis* (*1*), enhanced typing by SSU rRNA nested PCR–restriction fragment length polymorphism with *Ssp*I and *Vsp*I (*1*,*5*) was used for all isolates submitted to the UK Cryptosporidium Reference Unit during July and August. Samples from 23 cases (22 primary and 1 secondary) with rabbit genotype profiles were identified by visualization of 472-, 267-, and 109-bp bands generated by digestion with *Ssp*I (*1*). All case-patients lived in the area affected by the water supply incident and had onset dates consistent with exposure by drinking water consumption or by person-to-person spread. All 23 samples were homologous to AY120901 and AY273771 (Appendix Table). Of the other 11 samples, 6 were not confirmed by IFAT or PCR, 2 were *C*. *hominis*, 1 was *C*. *parvum*, and 2 were not typeable.

Sequences of the heat shock protein (HSP) 70 gene (*8*) and, to identify subtype family, the 60-kDa glycoprotein (gp60) gene (*9*) were determined for 7 water isolates and the rabbit and 9 outbreak case isolates. All HSP70 sequences were homologous with AY273775 from a rabbit in the Czech Republic (*7*) (Appendix Table). One water sample, the rabbit sample, and 8 human samples amplified the gp60 gene. These sequences were homologous with each other, but distinct from those published for *C*. *hominis* (subtype family I), *C*. *parvum* (subtype family II), *C*. *meleagridis* (subtype family III), and *C*. *fayeri* (subtype family IV) (*10*). Each rabbit genotype isolate had 18 TCA (serine) tandem repeats in the gp60 microsatellite region. We propose subtype family Va, subtype A18 for these isolates. This subtype differs from the rabbit genotype previously identified in a human in the United Kingdom (*1*) (subtype VaA22) (GenBank accession no. EU437420) and from rabbits in the Czech Republic (subtype VbA19) and China (subtype VbA29). Sequences generated during this study have been deposited in GenBank under accession nos. FJ262724–FJ262734.

Six additional persons infected with *Cryptosporidium* rabbit genotype were identified in August by testing of 394 stool samples from diarrheic patients; samples were routinely submitted for typing in July and August. All persons had onset dates inconsistent with the affected period and were from other regions. This finding may indicate a low background level of rabbit genotype cases. However, the prevalence is currently unknown.

The *Cryptosporidium* rabbit genotype has been identified as the etiologic agent in an outbreak of diarrheal disease and should be considered a human pathogen. Further studies commissioned by the Drinking Water Inspectorate (England and Wales) and funded by the Department of Environment, Food and Rural Affairs UK are underway.

## Supplementary Material

Appendix Table Location of nucleotide differences in the partial small subunit rRNA and heat shock protein 70 genes between Cryptosporidium hominis and the rabbit genotype*
