# Supplementary material for: Cryptosporidium Rabbit Genotype, a Newly Identified Human Pathogen
Source: Emerg Infect Dis. 2009 May;15(5):829–30. doi: 10.3201/eid1505.081419 (PMC2687022; doi:10.3201/eid1505.081419)
Supplement: Appendix Table — Location of nucleotide differences in the partial small subunit rRNA and heat shock protein 70 genes between Cryptosporidium hominis and the rabbit genotype* [file 08-1419_appT-s1.pdf]

Appendix Table. Location of nucleotide differences in the partial small subunit rRNA and heat shock protein 70 genes between *Cryptosporidium hominis* and the rabbit genotype*

Isolate (GenBank accession no.)	Location of nucleotide differences in the partial small subunit rRNA gene (nt 618–681)
C. hominis (AY204228)	TAATTTATATAAAATATTTTGATGAATATTTATATAATATTAACATAATTCATATTACTATTTTTTTTTTAGTATAT
Rabbit genotype (AY273771)	............T..........AG..–.......
Rabbit genotype (AY120901)	............T..........AG..–.......
Rabbit genotype from rabbit sample #17211	............T..........AG..–.......
Rabbit genotype from water sample #17200	............T..........AG..–.......
Rabbit genotype from human sample #17330	............T..........AG..–.......
	Location of nucleotide differences in the partial heat shock protein gene (nt 716–792)
C. hominis (XM_661662)	GACTCGTGGAATTCTGTGTACAAGATTTCAAGAGAAAGAATAGAGGTATGGATTTAACTTCAAATGCTAGAGCTTTA
Rabbit genotype (AY273775)	.......A...
Rabbit genotype from rabbit sample #17211	.......A...
Rabbit genotype from water sample #17200	.......A...
Rabbit genotype from human sample #17330	.......A...

*Dots indicate nucleotide identity with the C. hominis sequence from GenBank, and dashes indicate nucleotide deletions.
